# Calvarial Syphilitic Osteomyelitis as a Rare Manifestation of Secondary Syphilis in an Immunocompetent Patient: A Rare Diagnostic Challenge

**DOI:** 10.7759/cureus.83516

**Published:** 2025-05-05

**Authors:** Fatima Zeineddine, Abbas Rachid, Celestie Yaacoub, Rim Awada, Marwan Haddad

**Affiliations:** 1 Radiology, Lebanese University, Beirut, LBN; 2 Internal Medicine, Lebanese University, Beirut, LBN; 3 College of Medicine, Lebanese University, Beirut, LBN

**Keywords:** atypical secondary syphilis, calvarial bone osteomyelitis, mri imaging, skull osteomyelitis, syphilis

## Abstract

Syphilitic osteomyelitis of the skull is a rare manifestation of syphilis, typically associated with tertiary or congenital stages, and is exceptionally uncommon during secondary syphilis. We report the case of a 27-year-old immunocompetent, HIV-negative male who presented with persistent headaches and localized frontal scalp tenderness. Magnetic resonance imaging (MRI) revealed two calvarial lesions with adjacent soft tissue and meningeal enhancement. Serological testing confirmed secondary syphilis, while other sexually transmitted infections, including HIV, hepatitis B/C, *Neisseria gonorrhoeae*, and *Chlamydia trachomatis*, were excluded. Despite normal cerebrospinal fluid (CSF) findings, Venereal Disease Research Laboratory (VDRL) testing and HIV polymerase chain reaction (PCR) assays on CSF were not performed due to financial limitations. The patient was treated with a 14-day course of ceftriaxone, leading to complete symptom resolution and radiologic improvement. This case highlights the importance of including syphilitic osteomyelitis in the differential diagnosis of atypical cranial lesions, particularly in at-risk patients. It emphasizes the diagnostic value of MRI in such presentations.

## Introduction

Syphilis is a sexually transmitted infection (STI) that is caused by the spirochetal bacteria *Treponema pallidum*. It is a systemic disease that can affect many organs, leading to various symptoms. Bone involvement is a rare manifestation of syphilis [1]. Syphilis can present as primary, secondary, tertiary, or congenital. Bone lesions are associated with congenital or tertiary syphilis and, although infrequent, can also develop in secondary syphilis. The most common sites of bone involvement include the tibia, skull, sternum, and clavicles [2].A recent systematic review examining bony manifestations in secondary syphilis identified only 36 reported cases of bone involvement between 1964 and 2014 [3]. However, an increasing number of case reports describing syphilitic osteomyelitis in the early stages of the disease, particularly among individuals living with HIV, suggests that bone involvement may be a more common and underrecognized manifestation of syphilis than previously thought [4].

Hematogenous dissemination leads to bacterial depositions in the periosteum, Haversian canals, and medulla of the bones. The classical appearance in radiographs and CT is worm-eaten bone and adjacent sclerosis; however, less frequently, osteolytic lesions can be seen. MRI shows signal changes in the bone marrow, enhancement of the adjacent periosteum and dura, and adjacent soft tissue inflammation [2]. While established guidelines exist for the treatment of primary, secondary, tertiary, and congenital syphilis, there are currently no specific recommendations for managing syphilis with bone involvement [1]. Both intramuscular (IM) and intravenous (IV) penicillin have been used, but treatment durations vary widely. Reported regimens include 2 to 3 weeks of IV penicillin G for osteitis in early syphilis, three weeks of IM benzathine penicillin, or up to six weeks of IV penicillin or doxycycline for cases associated with later stages of the disease [4,5]. This report presents a rare case of calvarial osteomyelitis in the context of secondary syphilis, successfully treated with a 14-day course of intravenous and intramuscular ceftriaxone (1g daily), underscoring the potential efficacy of ceftriaxone in managing syphilitic bone disease.

## Case presentation

A 27-year-old Lebanese male patient presented to the hospital with the main complaint of severe headache with tenderness in the left frontal region. He noticed the headache two weeks before the presentation. There was no history of trauma, no documented fever, and no other symptoms associated. 

**Figure 1:**
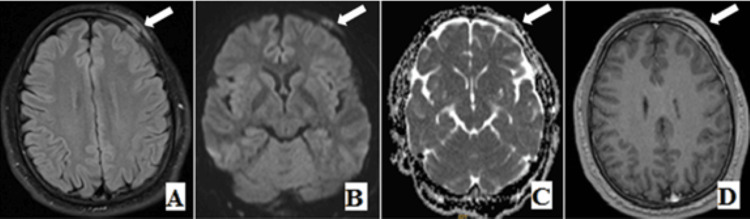
Axial T2/FLAIR, axial DWI/ADC Map (B,C), and axial gadolinium enhanced T1(D) showing T2/FLAIR hyperintense signal of the skull lesion in the left frontal bone and T2/FLAIR hyperintense soft tissue correlated with restriction on diffusion and enhancing bone, underlying meninges and overlying soft tissue (white arrow). DWI: Diffusion-weighted imaging, ADC: Apparent diffusion coefficient

He consulted a neurosurgeon for his problem, and an MRI brain was ordered to rule out any organic etiologies. Imaging shows the presence of a calvarial lesion in the left frontal bone measuring 1.5cm and showing high T2 signal intensity with restriction on diffusion and enhancement post gadolinium administration, associated with enhancement of the adjacent periosteum, dura, and adjacent soft tissue. A similar but slightly smaller (~1cm) calvarial lesion is also noted in the left parietal bone (Figure 1). Neoplastic and infectious causes were initially considered.

Consequently, the patient was referred to the infectious disease clinic for further evaluation, including a detailed medical history, clinical exam, and further laboratory studies. The patient reported having had sexual intercourse with anonymous male partners. He had never experienced symptoms suggestive of syphilis or been diagnosed with syphilis in the past.

On physical examination, the patient was hemodynamically stable with no signs of systemic illness. Pulmonary auscultation revealed good bilateral air entry without adventitious sounds, and heart sounds were regular without murmurs. No skin rashes, mucosal lesions, or lymphadenopathy were noted. Neurological examination was intact, with no focal deficits or signs of increased intracranial pressure. Notably, palpation of the scalp revealed localized tenderness over the affected area, although there was no visible swelling, erythema, or fluctuance. Laboratory investigations demonstrated an elevated C-reactive protein level, suggesting an inflammatory process. Complete blood count, liver function tests, and renal function parameters were all within normal limits.

Given the patient's history, being a man who has sex with men (MSM), which is associated with a higher risk for sexually transmitted infections (STIs), a comprehensive STI screening panel was performed. Tests for HIV, hepatitis B, and C, *Neisseria gonorrhoeae*, and *Chlamydia trachomatis* were all negative. Syphilis was the only positive finding, with significantly elevated *Treponema pallidum* hemagglutination assay (TPHA) titers (1:5120) and a reactive venereal disease research laboratory (VDRL) test result (28.19, reference value: >1.0 considered reactive). Although cerebrospinal fluid (CSF) analysis showed a normal cell count, CSF VDRL testing was not performed due to financial constraints. Based on the clinical history, physical examination, and serologic findings, a diagnosis of secondary syphilis was established. Since neurosyphilis could not be definitively excluded, the patient was treated with a 14-day course of ceftriaxone (1 g daily, initially intravenous then intramuscular). At a three-week follow-up visit, the patient showed marked clinical improvement, with complete resolution of left frontal tenderness.

**Figure 2:**
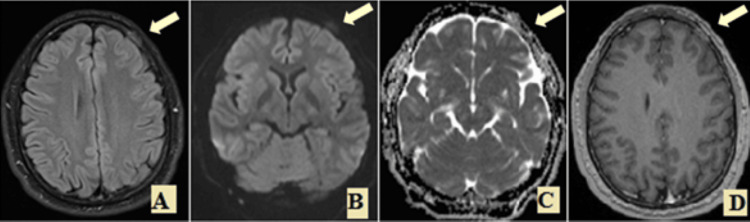
Axial T2/FLAIR, axial DWI/ADC Map (B,C), and axial gadolinium enhanced T1(D): a follow-up three months later, shows diminution of the intra-/extra-osseous soft tissue process and decrease of the meningeal enhancement as well as the contrast enhancement and the diffusion restriction (arrow). DWI: Diffusion-weighted imaging, ADC: Apparent diffusion coefficient

A follow-up MRI three months after the initial imaging demonstrated a diminution of the intra-/extra-osseous soft tissue process and a decrease of the meningeal enhancement, as well as the contrast enhancement and diffusion restriction (Figure 2). Follow-up laboratory studies showed a normal c-reactive protein (CRP); however, the VDRL test demonstrated a positive result (26.02). The TPHA test was not available. At 12 months, a repeated VDRL test showed a significant decrease to 5, indicating a favorable serologic response to treatment.

## Discussion

Calvarial lesions are usually discovered incidentally on radiologic imaging or manifest with symptoms. A variety of neoplastic and nonneoplastic (traumatic, infectious, metabolic…) conditions may involve the skull and pose a diagnostic challenge. The differential diagnosis can be narrowed using the combination of clinical information and the imaging features of skull lesions [6]. One of the causes of skull lesions is Syphilis, which is considered a great imitator that can influence the skeletal structures and mimic chronic non-specific osteomyelitis, primary bone tumor, metastatic cancer, multiple myeloma, eosinophilic granuloma, or any other type of bone lesion [7]. Various bones can be involved in syphilitic osteomyelitis, including the skull, clavicle, tibia, humerus, ulna, and radius [8]. The bone marrow and periosteum have an abundant blood supply, making these structures a good reservoir of infection. Consequently, “*Treponema Pallidum*” is borne to all parts of the body via the bloodstream, and the invasion of the periosteum or bone manifested by osteomyelitis is to be expected [7].

Magnetic resonance imaging has an essential role in providing a non-invasive technique to assess the skull lesion by demonstrating the presence of marrow space involvement, periosteal process, and degree of meningeal and intracranial extension more completely than CT. The skull lesion of our patient had similar characteristics to the MRI findings presented in the few reports of acquired syphilitic osteomyelitis of the calvaria published in clinical literature. The lesions show focal enhancement with adjacent enhancing soft-tissue abnormality in the scalp, as our case showed [9].

Syphilitic osteomyelitis can involve various bones, including the skull, clavicle, tibia, humerus, ulna, and radius. Management primarily relies on antibiotic therapy, with benzathine penicillin being the first-line treatment, and ceftriaxone serving as an effective alternative, particularly in cases of penicillin allergy or where daily intramuscular injections are more feasible. While most cases respond well to medical therapy alone, surgical intervention may be performed when there is diagnostic uncertainty or suspicion of a neoplastic process. Indeed, osteomyelitis of the skull as the sole and initial presenting manifestation of syphilis is extremely rare, especially in immunocompetent individuals and in regions where syphilis is not highly endemic, like in Lebanon [6]. MRI also follows the progression of the bone lesion and its response to treatment. Studies show that although symptomatic relief after therapy is typically rapid, the calvarial lesions resolve more slowly and can persist for up to 7-11 months. Furthermore, when proper therapy is given, there can be complete resolution of lesions on imaging, with little residual abnormality [9].

Recent epidemiological data showed a resurgence of syphilis in the current decade, which coincided with an increase in promiscuous homosexuality. A recent cross-sectional study carried out in a dermatology outpatient department among 560 cases diagnosed as having sexually transmitted infections revealed a rising trend of all sexually transmitted infections (STIs), including syphilis, from 2011 onward, and syphilis was the most common STI among males having sex with males [10]. Therefore, syphilis should be reintroduced into the daily routine of radiologists as this “great imitator” is challenging and may present with uncommon manifestations. Although rare, osteomyelitis can be found in the context of secondary syphilis, so it must be on the list of differential diagnoses of osteolytic lesions [2].

## Conclusions

In conclusion, syphilitic osteomyelitis of calvarium, although rare, should be considered in patients presenting with focal cranial symptoms and risk factors for sexually transmitted infections, particularly in the men who have sex with men (MSM) population. MRI serves as a vital tool in the non-invasive diagnosis and follow-up of these lesions, helping differentiate them from neoplastic or other infectious processes. Early diagnosis, supported by serologic testing and imaging, allows for prompt initiation of antibiotic therapy, often leading to full clinical recovery and radiologic resolution. This case further underscores the necessity of maintaining a high index of suspicion for syphilis in atypical presentations to avoid misdiagnosis and unnecessary invasive interventions.
